# Editorial: Bottom-Up and Top-Down: Molecules and Circuits That Underlie Chemosensory Behaviors

**DOI:** 10.3389/fncel.2021.729791

**Published:** 2021-08-13

**Authors:** Pablo Chamero, Shaina M. Short, Jeremy C. McIntyre, Julian P. Meeks, Markus Rothermel

**Affiliations:** ^1^CNRS, IFCE, INRAE, Université de Tours, Nouzilly, France; ^2^Neurobiology Department, University of Utah School of Medicine, Salt Lake City, UT, United States; ^3^Department of Neuroscience, Center for Smell and Taste, University of Florida, Gainesville, FL, United States; ^4^University of Rochester School of Medicine and Dentistry, Rochester, NY, United States; ^5^Institute for Physiology and Cell Biology, University of Veterinary Medicine Hannover, Foundation, Hanover, Germany

**Keywords:** neuromodulation, behavior, physiology, social cues, chemosensation

This Research Topic highlights recent advancements in our understanding of how genes and neural circuits in the brain shape complex natural behaviors that are evolutionary conserved and essential for survival across species. Like any cortical area, including visual and somatosensory cortices, the chemosensory brain is strongly affected by attention, expectation, and perceptual tasks mainly mediated by so-called “top-down” inputs (Gilbert and Sigman, 2007; McIntyre et al., 2017). “Bottom-up” and “top-down” networks converge in early sensory areas and higher brain regions. Top-down systems signal risk, reward, novelty, effort, and social cooperation (Richardson and DeLong, 1986; Krichmar, 2008; Rutledge et al., 2015; Schultz, 2015). These systems provide the basis for higher cognitive functions like attention (Thiele and Bellgrove, 2018), decision-making (Rutledge et al., 2015), emotion (Wang and Pereira, 2016), and goal-directed behavior (Hirayama et al., 2014), which are thought to result from the interaction between top-down and higher brain areas (Mesulam, 1998). Through top-down processes, factors like experience, motivation and expectation, i.e., the general “brain state,” affect sensory input analysis and, thereby, our internal representation of the world (Manita et al., 2015).

How the brain balances critical extrinsic, bottom up and intrinsic, top-down information to enable adaptive behaviors is currently a major topic in neurobiology. All organisms rely on chemosensory systems to seek food, communicate, and avoid hazards and predators. In turn, internal brain states modulate the detection of external chemosensory stimuli and chemosensation is shaped by hunger, the drive to procreate, hormones, stress and fear. This requires neural circuits to adapt depending on the behavioral needs (wakefulness/sleep state, arousal, learning, metabolic demands, reproduction state, etc.). Therefore, chemosensation represents an excellent model system to investigate how the brain processes extrinsic and intrinsic information (Lizbinski and Dacks, 2017; Brunert and Rothermel, 2021). Since chemosensory information can reach cortical areas without thalamic involvement, the organization of these bottom-up pathways is at least partially different compared to other sensory systems. On the other hand, the vast majority of its top-down projections are not chemosensory brain-specific and innervate a broad range of sensory cortical and subcortical areas. The link between chemosensory processing and behavior has been investigated in great detail in both vertebrates and invertebrates. By making use of an advanced genetic toolset, mechanisms adapting chemosensory processing and perception to internal states such as hunger and reproductive state have been investigated in great detail in invertebrates (Sayin et al., 2018). In mice, olfactory cues such as pheromones have been shown to elicit specific social forms of behavior (Stowers and Liberles, 2016) and hippocampal top-down projections to sensory centers have been shown to match place information to odor memory, thereby linking internal brain state, top-down activity, and memory processes (Aqrabawi and Kim, 2018).

Work from many labs has demonstrated that the activity of neurons at any stage of chemosensory information can be modulated to optimize stimulus processing and behavioral outputs. This Research Topic captures the current progress on our understanding of how changes in chemosensory neural circuits can have significant behavioral consequences.

This Research Topic comprises a wide variety of articles contributing to current views and understanding of different bottom-up and top-down systems: pioneering researchers in the field use state-of-the-art tools such as, genetically encoded neurotransmitter indicators and odor plume concentrations measurments and cover a wide spectrum of methods that range from genetics to behavioral analysis. The reader will find several articles related to the architecture and function of neural circuits involved in social signal processing. Transgenic approaches that partially eliminate sensory function in the vomeronasal organ are used to show that social behavior in female mice results from interactions between intrinsic mechanisms in the vomeronasal system and experience-dependent plasticity (Trouillet et al.). In order to explore whether motherhood alters sensory processing of pup-derived chemosignals (Navarro-Moreno et al.) analyzed the expression of immediate early genes in the vomeronasal organ and centers of the olfactory and vomeronasal brain pathways in virgin and late-pregnant females. They found changes in sensory processing with pregnancy in both the main as well as the accessory olfactory system. Finally, Chu et al. used mass staining, calcium imaging, and intracellular recordings to characterize the morphological and physiological properties of the male-specific macroglomerular complex (MGC) neurons of the lateral tract in the moth antennal lobe. These pheromone-sensitive projections neurons could mediate fast control of hard-wired behavior like e.g., odor tracking.

In behavioral studies, odor tracking in mammals and odor plumes were investigated in a publication from David Gire's lab. In order to investigate how plume dynamics impact early odor representation in the mouse olfactory system (Lewis et al.) measured wide-field GCaMP6f signaling from the dendrites of mitral and tufted cells in head-fixed animals in response to naturally fluctuating odor plumes. They found that across flow conditions odor dynamics are a major driver of activity in many glomerular networks. In another study, activation of the dopamine receptor D1 was found to potentiate feedforward excitation in the olfactory bulb, enhancing mitral tufted cell output and sensitivity to odor stimuli (Liu). The results provide a mechanistic basis for the functional roles of dopamine in modulating odor detection and discrimination. In order to determine how local cholinergic signaling impacts basal forebrain output pathways that participate in top-down regulation (Hanson et al.) utilized fiber photometry and the genetically encoded acetylcholine indicator GAChR2.0 to define temporal patterns of cholinergic signaling in the basal forebrain during olfactory-guided, motivated behaviors, and learning. Their results suggest that cholinergic tone in the basal forebrain changes rapidly to reflect reward-seeking behavior and positive reinforcement and may impact downstream circuitry that modulates olfaction. The role of neuromodulators was also investigated in non-neuronal cell types. Confocal calcium imaging and immunohistochemistry in mouse olfactory bulb slices provide insight into calcium signaling evoked by norepinephrine in astrocytes (Fischer et al.). At the periphery, genetic control of chemosensory receptors is critical for the intial detection of odors, yet the choice of odorant receptors is still not fully known. The role of genetic background itself on olfactory receptor expression was investigated by Leme Silva et al. This study analyzed the expression of the OR gene Olfr17 (also named P2) in different genomic contexts and show that genetic variations in *cis* regulatory regions can lead to differential DNA methylation frequencies in these OR gene alleles. Expression of the *OR* alleles is largely affected by the genetic background as well as epigenetic modifications.

All together, this Research Topic highlights data from different model organisms, neuronal as well as glia cells and incorporates genetic, physiological, and behavioral approaches to offer new perspectives about the complex interaction of bottom-up and top-down systems.

## Author Contributions

MR wrote the first draft of the manuscript. All authors contributed to manuscript revision, read, and approved the submitted version.

## Conflict of Interest

The authors declare that the research was conducted in the absence of any commercial or financial relationships that could be construed as a potential conflict of interest.

## Publisher's Note

All claims expressed in this article are solely those of the authors and do not necessarily represent those of their affiliated organizations, or those of the publisher, the editors and the reviewers. Any product that may be evaluated in this article, or claim that may be made by its manufacturer, is not guaranteed or endorsed by the publisher.
